# Understanding Aging Mechanisms in the Context of UV Irradiation of a Low Fouling and Self-Cleaning PVDF-PVP-TiO_2_ Hollow-Fiber Membrane

**DOI:** 10.3390/membranes12050538

**Published:** 2022-05-21

**Authors:** Emma Roubaud, William Maréchal, Olivier Lorain, Lina Lamaa, Laure Peruchon, Cédric Brochier, Julie Mendret, Jean-Pierre Mericq, Stephan Brosillon, Catherine Faur, Christel Causserand

**Affiliations:** 1 Laboratoire de Génie Chimique, Université de Toulouse, CNRS, INPT, UPS, CEDEX 9, 31062 Toulouse, France; roubaud.emma@yahoo.com; 2 Polymem, 3 Rue de l’Industrie, 31320 Castanet-Tolosan, France; w.marechal@polymem.fr (W.M.); o.lorain@polymem.fr (O.L.); 3 Brochier Technologies, 90 Rue Frédéric Fays, 69100 Villeurbanne, France; lina.lamaa@brochiertechnologies.com (L.L.); laure.peruchon@brochiertechnologies.com (L.P.); cedric.brochier@brochiertechnologies.com (C.B.); 4 Institut Européen des Membranes, IEM, UMR 5635, Université de Montpellier, ENSCM, CNRS, Place Eugène Bataillon, CEDEX 5, 34095 Montpellier, France; julie.mendret@umontpellier.fr (J.M.); jean-pierre.mericq@umontpellier.fr (J.-P.M.); stephan.brosillon@umontpellier.fr (S.B.); catherine.faur@umontpellier.fr (C.F.)

**Keywords:** membrane stability, radical oxidation, photocatalytic material

## Abstract

In the context of designing a photocatalytic self-cleaning/low-fouling membrane, the stability of PVDF-PVP-TiO_2_ hollow-fiber membranes under UV irradiation has been studied. The effect of irradiation power, aqueous environment composition and fouling state on the properties of the membranes has been investigated. With this aim, SEM observations, chemical analysis and tensile strength measurements have been conducted. The results indicate that pristine membranes that undergo UV irradiation in ultra-pure water are significantly degraded due to attacks of OH° radicals. However, when methylene blue, used as a model pollutant, is introduced in the aqueous environment, OH° radicals preferentially react with this molecule rather than the membranes, successfully preserving the original properties of the latter. The presence of an adsorbed BSA layer (pre-fouling by immersion) on the surface of the membrane delays membrane aging, as the BSA layer is degraded by radicals instead of the membrane material. The degradation of the BSA layer also validates the self-cleaning properties of the membrane. However, when membranes are pre-fouled by filtration of a 2 g/L BSA solution, delay to aging is less. This is because OH° radicals do not reach BSA molecules that are trapped inside the membrane pores, and therefore react with the membrane material.

## 1. Introduction

Membrane processes applied to wastewater treatment have experienced remarkable growth the past fifteen years because they are able to cope with water qualities and flow fluctuations that conventional processes cannot handle [1]. However, fouling issues, inherent to any membrane process, have a negative impact both financially (energy and chemical cleaning costs, operation interruption) and environmentally (cleaning treatment). Previous works aiming to overcome this problem have focused on optimizing membrane module configurations or operating conditions [1,2]. In this context, the development of low-fouling and self-cleaning membranes constitutes a major paradigm shift. Solutions to limit membrane fouling include surface modifications (grafting) which reduce membrane interaction with natural organic matter [3] and/or increase hydrophilic properties of the membrane material [4,5]. Another solution is the use of forward osmosis [6]. To this day, none of the proposed solutions to limit this fouling has proved to be truly satisfactory. These solutions induce a loss of the polymer chemical properties, accelerate aging of the membrane, have high energy costs, cause a drop in the productivity of the plant or induce the high consumption of chemicals [7,8,9].

Polyvinylidene difluoride (PVDF) membranes are commonly used in ultrafiltration, microfiltration and pervaporation processes since this polymer presents a priori high chemical, thermic and mechanical resistance making these membranes suitable for frequent chemical cleaning and backwashing operations [10]. Ongoing research includes a crystalline photocatalyst in PVDF membranes as a way to provide them with antifouling and self-cleaning proprieties [10,11,12,13,14]. Irradiating the photocatalyst with UVA light creates a positive hole at the valence band and an electron at the conduction band. This promotes two mechanisms: (1) a photo-induced super-hydrophilicity mechanism that limits membrane fouling as most of the foulants are hydrophobic [15] and (2) the formation of highly oxidative hydroxyl radicals (OH°) through oxidation and reduction reactions near the photocatalyst surface. These radicals degrade organic matter, responsible for fouling, and provide the membrane with self-cleaning properties [16]. The most commonly used photocatalyst is TiO_2_, in rutile and anatase crystalline forms, where illumination with light of less than 385 nm promotes a redox environment [17].

In the same way that conventional sodium hypochlorite washing ages membranes [18,19], there is a risk that hydroxyl radicals, produced through photocatalysis, deteriorate the morphological, mechanical and chemical properties of a photo-catalytic membrane [17,20]. This concerns not only the backbone polymer of the membrane (PVDF) but also hydrophilic additives (such as polyvinylpyrrolidone PVP) which are, in the majority of cases, more sensitive to radical oxidation [18]. It is therefore crucial to understand the mechanisms responsible for membrane aging, in order to find a balance between the removal of fouling organic matter and the protection of membrane material. Most studies regarding photocatalytic membranes focus on the characterization of the self-cleaning effect, while only a few focus on the stability of the membrane material during UV irradiation, particularly when exposed long-term [21,22,23]. These studies all report detrimental damage to membrane structural morphologies and chemical characteristics upon UV exposure, with the apparition of cracks and fractures on the outer surface of the membrane. Nevertheless, polymer degradation still requires further study under various irradiation conditions (amount of UV received, composition of the medium surrounding the membranes, static or dynamic environment, etc.).

In this present work, a study of the aging of a PVDF-PVP-TiO_2_ hollow-fiber (HF) membrane under UV irradiation was carried out. Accelerated aging is first achieved by subjecting HFs to high amounts of UV radiation in an ultra-pure (UP) water medium, which represents the most detrimental condition. The experimental conditions of the follow-up experiments aim to mimic real-life working conditions that are expected to be less detrimental for membranes.

Membrane degradation was first characterized at various UV irradiation powers on pristine HFs in an UP water static environment. Then, the effect of the presence of methylene blue (MB) a model pollutant in the aqueous environment on membrane properties evolution was studied. Finally, membranes were pre-fouled with bovine serum albumin (BSA) by using two different methods and aged as in the previous experiments in order to (1) assess the effect of the presence of fouling on membrane degradation and (2) validate the self-cleaning properties of the membranes.

## 2. Materials and Methods

### 2.1. Hollow-Fiber Membrane Preparation

HF membranes have been prepared with NIPS (Non-solvent Induced Phase Separation) process. First, a blend of polymer and additive, called dope solution, was prepared. PVDF polymer (PVDF resin Kynar^®^ from Arkema) was dissolved in a stirred and heated reactor: N-Methyl-2-Pyrrolidone (NMP) was used as a solvent for the polymer dissolution and PVP was added as pores former and hydrophilic agent. Prior to the dope solution fabrication, TiO_2_ nanoparticles were dispersed in the NMP by stirring and sonication to avoid aggregates. HFs were fabricated using a spinning line. Dope solution was extruded in a hollow fiber shape through a specific spinneret: dope solution was fed around a needle through which a bore liquid, made of solvent/non-solvent mixture, was flowing to form the lumen of the fiber. HFs were then immerged in a coagulation tank filled with non-solvent, water in our case, and rinsed to remove solvent and additives in a washing step before being collected on spoons. Finally, asymmetric UF HFs were obtained with external diameter 0.76 mm and internal diameter = 0.43 mm.

### 2.2. Chemicals

Methylene blue (MB, reference 229801000, high purity biological stain, CAS-7220-79-3) and bovine serum albumin (BSA, reference 134731000, Fraction V, purity 96–100%) were purchased from Acros Organics (Geel, Belgium). Solutions were prepared with ultra-pure (UP) water (18 MΩ.cm at 25 °C).

### 2.3. BSA Adsorption-Filtration

Two methods were used to deposit BSA on HFs surface prior to irradiation. On one hand, HFs were soaked in a 10 g/L BSA solution for 72 h in order for the BSA to be adsorbed on HFs surface (HFs noted “BSA_ads_”). On the other hand, HFs were put in a homemade PVC module and were fouled by filtration of 5 L of a BSA solution (2 g/L) at a constant pressure of 1 bar for 4 h. At the end of the BSA filtration, the module was opened and HFs (noted “BSA_f_”) harvested for irradiation and further analyses.

### 2.4. Pure Water Permeability

For the measurement of pure water permeability on the pristine membrane and on the BSA pre-adsorbed samples, 30 cm-long homemade modules were prepared containing 30 fibers. For the membranes that were first subjected to the BSA filtration, the pure water permeability was measured just before opening the module and collecting the fibers.

In each case, intrinsic membrane permeability was determined by filtration of UP water through the module at different transmembrane pressures in dead-end mode (0.2, 0.4, 0.6, 0.8, 1.0 and 1.2 bar).

Pristine HFs pure water permeability (Lp) was on average 283 L/h/m²/bar and decreased to 189 L/h/m²/bar for BSA_f_ HFs. No significant Lp modification was observed on BSA_ads_ HFs compared to pristine HFs.

### 2.5. Membrane Aging

About 150 strands of HFs membranes were disposed side-by-side so as to create a 20 cm length curtain. The curtain was taped on a hollow PVC structure allowing it to stand vertically. The structure was placed in a 5 L glass beaker filled with UP water or MB solution. The volume of fibers represented 0.26 % of the soaking solution. HFs were then irradiated on both sides with UVA light sources (LED coupled to an optical fiber bundle, 365 nm) placed at a distance of about 1 cm from the curtain. The LED is powered by a dimmable power supply. The electric current can vary between 50 and 1000 mA. The electric current is proportional to the radiant flux of the LED. The more we increase the luminous flux, more the transmission of light at the surface of the optical fiber bundle increases and therefore the surface irradiance increases (value in µw/cm^2^).

A control experiment using a similar structure without UV irradiation was also set up in parallel.

HF samples were periodically collected on the curtain for the duration of the experiments (384 h/16 days).

### 2.6. Morphology Observation

The morphologies of the surface and the cross-section of the membranes were examined using scanning electron microscopy (SEM) on a Hitachi TM-1000 table-top microscope (Hitachi, Tokyo, Japan) at magnifications of ×350, ×1300 and ×15,000. Membranes were fractured after being dipped for 15 s in liquid nitrogen in order to avoid the crushing of the fiber during the fracture. Each HF undergoes five cuts. Each cut was coated with a thin layer of gold by sputtering before observation. Membrane wall thickness was then measured on five locations on the cross-section SEM image obtained. A total of twenty-five measurements per HF were then conducted; the values shown in the figures below are accompanied by the standard deviation thus obtained.

### 2.7. Tensile Strength Tests

Elongation at break was determined with an Instron 3342 Series tensile strength test machine. Measurements were conducted on wet membrane samples at a 50 mm gauge length with a 150 mm/min strain rate. Elongation at break was calculated from the experimental stress–strain curves using Bluehill 3 software. The values reported were averaged on five tests for each data point.

### 2.8. ATR-FTIR

ATR-FTIR analysis was performed on a Thermo Nicolet 6700 (Waltham, MA USA) infrared spectrometer fitted with a DTGS detector and a diamond ATR crystal. Spectra were obtained by summation of 16 scans between 4000 and 400 cm^−1^ with a resolution of 4 cm^−1^. Spectral analysis was performed using the OMNIC^®^ software.

### 2.9. Soaking Medium Analysis

During irradiation of HFs, liquid samples were collected from the soaking medium. Fluorine concentration was analyzed by ion-exchange chromatography on a Dionex ICS 3000. Titanium concentration was analyzed by inductively coupled plasma atomic emission spectroscopy (ICP-AES) on a Horiba Ultima machine (Kyoto, Japan). MB concentration was analyzed by absorption spectroscopy on a Perkin Elmer UV-visible spectrometer (Waltham, MA, USA) at 670 nm after diluting the samples in order to obtain an absorption value between 0 and 2. The Beer–Lambert law was used to link the absorbance to the concentration. A calibration curve was produced with MB solutions of concentrations ranging between 1.3 × 10^−5^ mol/L and 1.3 × 10^−6^ mol/L, giving a linear equation of A = 63441 C and a determination coefficient R^2^ of 0.999.

## 3. Results and Discussion

### 3.1. Aging of HFs Soaked in UP Water during Irradiation–Effect of UV Irradiation Power

#### 3.1.1. Structural Degradation

**Figure 1:**
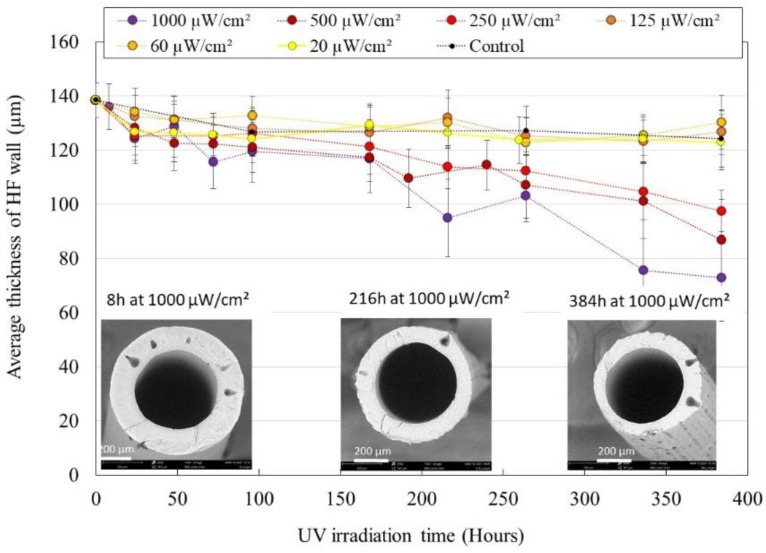
Average thickness of HFs’ wall after UV irradiation in UP water with various irradiation powers. Insert: SEM images of HFs irradiated at 1000 µW/cm² during 8 h, 216 h or 384 h (×350 magnification).

UV irradiated HFs samples were characterized to assess the degradation of their morphological properties. Figure 1 shows the evolution of average HF wall thickness during exposition to UV light at various irradiation powers. At an irradiation power of 250, 500 and 1000 µW/cm², a significant decrease in HFs’ wall thickness was observed, down to −50% for HFs irradiated at 1000 µW/cm² for 384 h. Measurements of HFs’ internal diameter showed no evolution during the whole experiment, meaning that the degradation happens on the external side of the HFs. At an irradiation power of 20, 60 and 125 µW/cm², no significant evolution of HFs’ wall thickness was observed when compared to the evolution of control HFs (membranes soaked in UP water without irradiation).

SEM images of the cut HFs showed the presence of frequent drop-like macrovoids inside the membrane wall (average length 54 ± 16 µm; average width 29 ± 5 µm). External layer degradation made those macrovoids clearly visible on the HFs outer surface (Figure 1, right SEM image). Cracks appeared running along the lines of emerging macrovoids, which is commonly observed in similar membrane aging studies [22,23,24].

**Figure 2:**
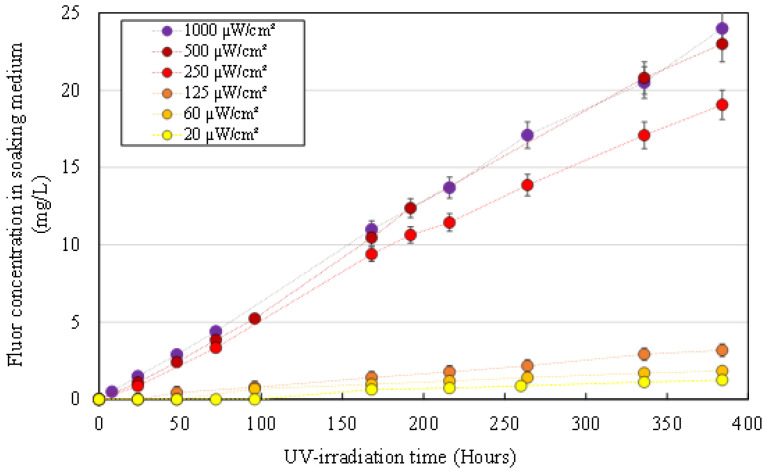
Fluorine concentration in UV irradiated HFs soaking medium for different irradiation powers.

PVDF degradation was confirmed through the analysis of fluorine concentration in the soaking medium of UV irradiated HFs. The fluorine concentration in the bath during UV exposition is presented in Figure 2. Significant increases of fluorine concentration were observed even at the lowest irradiation power. In liquid samples collected in the control beaker, fluorine was not detected through ionic chromatography analysis. 

The dehydrofluorination phenomenon, which is an ionic mechanism, is commonly observed on PVDF membranes exposed to UV light and consists in the formation of a carbon-carbon double bond as a result of the elimination of hydrogen fluoride from the polymer [25,26]. This reaction causes PVDF to change color from white to brown [23], which was not observed in our HFs. Instead, the PVDF forming the structure of HFs was degraded, which is supported by the SEM images and measurements presented in Figure 1. This degradation doubtless happens through radical processes which include polymer chain scission, double bond formation, crosslinking and secondary oxidative reactions [27,28,29]. As PVP is known to be highly sensitive to radical oxidation, we can speculate that oxidation of the PVP component promoted the oxidation of the PVDF component, as it was the case for Prulho et al., with a polyethersulfone/PVP ultrafiltration membrane in contact with bleach water [18]. The complete degradation of the membrane structure material led to the release of entrapped TiO_2_ NP, which was deposited at the bottom of the soaking beaker (nature of the deposit was confirmed through ATR-FTIR analysis, see Appendix A). Titanium concentration in solution qualitatively increased during irradiation. As TiO_2_ NP tends to form clots, sampling could not be performed homogeneously and the graphical representation of titanium concentration during irradiation is not displayed here.

#### 3.1.2. Mechanical Properties Evolution

**Figure 3:**
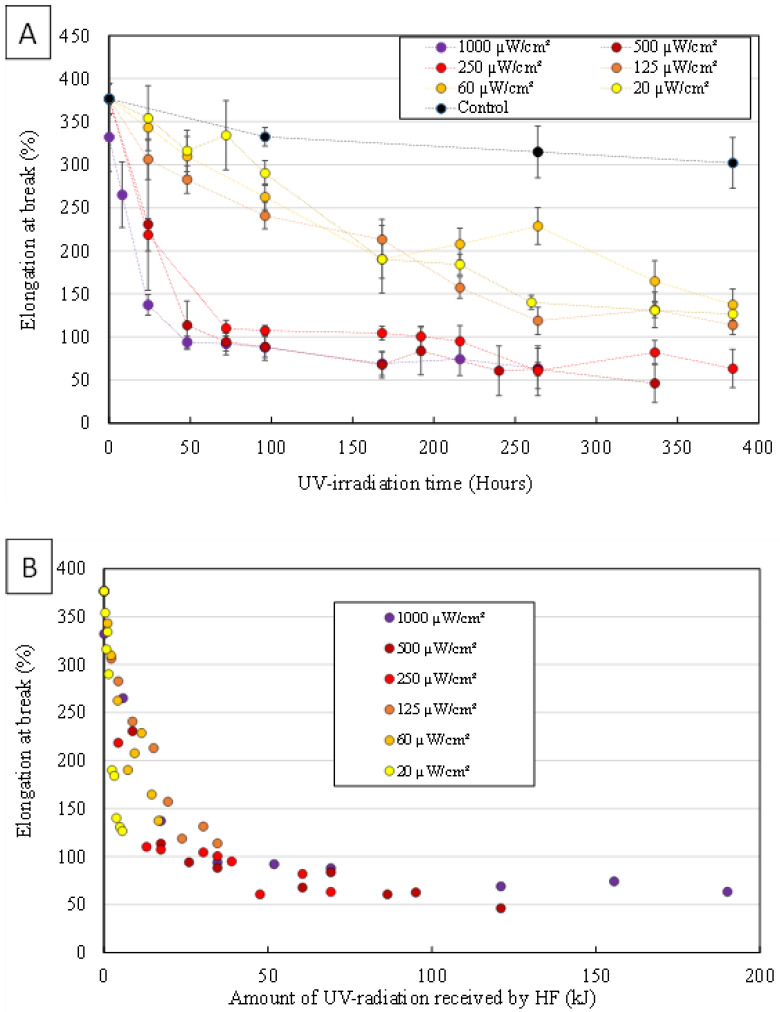
Elongation at break measured on UV irradiated HFs as a function of (**A**) the irradiation time or (**B**) the amount of UV radiation received.

Tensile strength tests were performed on UV irradiated HFs samples to assess the degradation of their mechanical properties. Figure 3A shows the effects of UV irradiation power and duration on elongation at break of HFs.

Elongation at break decreased significantly from the first hours of irradiation even at the lowest irradiation power (20 µW/cm²). At the highest irradiation power (1000 µW/cm²), after 336 h of UV exposure, HFs had become so brittle that it was not possible to correctly perform the tensile strength measurements. As resistance is closely related to membrane structure, elongation at break is a direct measure of membrane damage [17]. The reduced HFs’ wall thickness due to intense PVDF degradation, the macroporous structure caused by the emerging macrovoids and the possible embrittlement of the remaining polymer material resulted in deleterious effects on the mechanical strength of HFs [17,22,23,28,30]. This caused most HFs to lose their integrity and for this reason, aged HFs could not be properly characterized in terms of water permeability during the experiments.

Membrane material degradation seems to be positively correlated to irradiation power. Nevertheless, for all characterization techniques, results obtained with the lowest three irradiation powers are of very similar values, in the same way than results obtained with the highest three irradiation powers. These “groups” suggest that at an irradiation power of 250 µW/cm² or more, HFs’ mechanical, chemical and morphological degradation gets faster. Figure 1 shows that a significant decrease in HF wall thickness is only observed for HFs irradiated at 250 µW/cm² or more. The emergence of TiO_2_ NP originally entrapped inside HFs’ skin and their exposition to UV light as the membrane material gets degraded could further enhance degradation speed.

Figure 3B shows the effects of the amount of UV radiation received by HFs on elongation at break. As all data points follow the same envelope, we assume that the degradation mechanisms are the same regardless of the UV irradiation power used. The observed phenomenon only depends on the amount of UV radiation received by the fibers.

Thereby, in the other experiments presented in this study, the irradiation power used was 1000 µW/cm² in order to exacerbate the phenomena investigated. Under these conditions, the results obtained on pristine HFs soaked in pure water presented above (purple dots) were then compared to both the results obtained when MB as model pollutant was added in the soaking medium, as well as to the results obtained with membranes subjected to pre-fouling (BSA_ads_ and BSA_f_ HFs) before immersion in pure water and irradiation.

### 3.2. Aging of HFs Soaked in MB Solution during Irradiation

HFs were irradiated at 1000 µW/cm² for 384 h in an MB solution with an initial concentration of 4.5 × 10^−5^ mol/L. Irradiated HFs were characterized by the measurement of their average wall thickness and elongation at break. MB concentration in the soaking medium during irradiation was also measured. A control experiment with HFs soaked in MB solution without UV irradiation was also performed.

#### 3.2.1. Morphological Degradation

**Figure 4:**
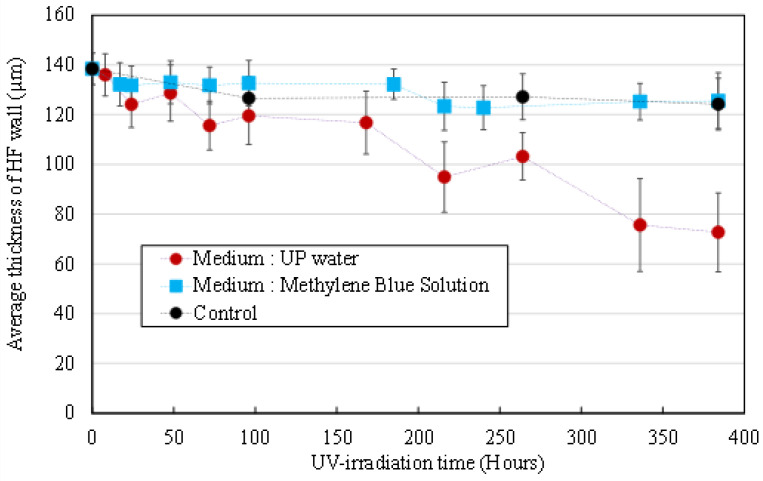
Average thickness of HFs’ wall after UV irradiation at 1000 µW/cm² in an MB solution (**blue squares**), in UP water (**red dots**). Control is non-irradiated HFs in am MB solution (**black dots**).

Figure 4 shows the evolution of the average HFs’ wall thickness during UV irradiation in an MB solution (blue squares) and UP water (red dots). For the whole duration of the experiment, no significant variation of wall thickness was observed for HFs irradiated in an MB solution (compared to the control non-UV irradiated HFs soaking in an MB solution, black dots), when a drastic decrease of wall thickness was observed on HFs irradiated in UP water. The presence of MB molecules in the aqueous medium successfully inhibits the alteration of the membrane material.

#### 3.2.2. Mechanical Properties Evolution

**Figure 5:**
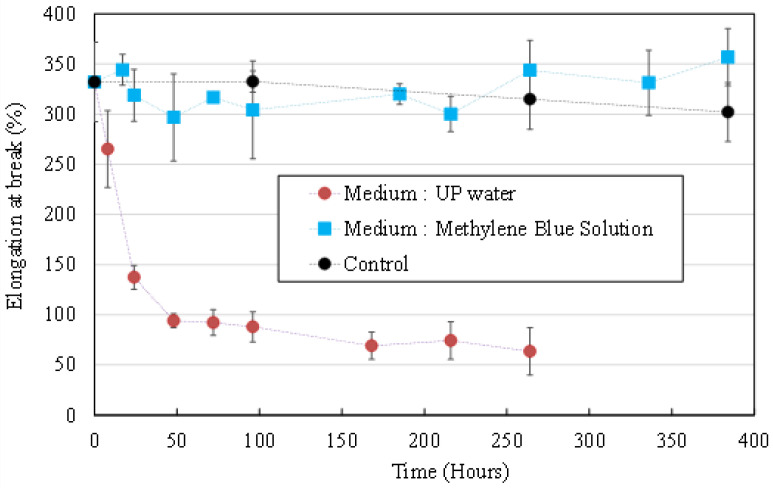
Elongation at break measured on HFs irradiated at 1000 µW/cm² in an MB solution (**blue squares**), in UP water (**red dots**). Control is non-irradiated HFs in an MB solution (**black dots**).

Figure 5 shows the evolution of the average HFs’ elongation at break during UV irradiation in an MB solution (blue squares) and UP water (red dots). As for average wall thickness, no significant variation of elongation at break was observed for HFs irradiated in an MB solution (compared to the control non-UV irradiated HFs soaking in an MB solution, black dots). The presence of MB molecules in the aqueous medium successfully inhibits the morphological and mechanical properties’ degradation that was observed on HFs that were irradiated in UP water.

#### 3.2.3. MB Concentration in the Aqueous Medium

**Figure 6:**
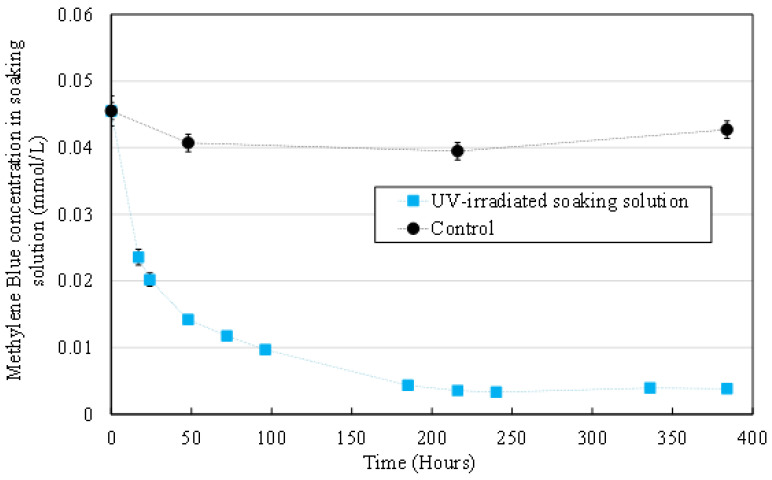
MB concentration in liquid samples collected from the soaking medium of irradiated HFs (**blue squares**) and non-irradiated control HFs (**black dots**).

MB concentration was measured by absorbance spectroscopy at 670 nm for liquid samples collected from the soaking medium of irradiated HFs (blue squares) and non-irradiated control HFs (black dots). Results are displayed in Figure 6.

An important decrease was observed for MB concentration in the soaking medium of UV irradiated HFs. This decrease cannot be attributed to direct MB photolysis under UV irradiation as it has been shown by Zhang et al., [31] for a similar wavelength and higher light irradiance (5.3 mW/cm² at λ = 360 nm) that MB was not degraded over 120 min. Moreover, no significant evolution was observed for MB concentration of the control experiment (non-irradiated control HFs). These results, associated with the fact that no significant evolution of the morphological and mechanical properties of HFs was observed (Figure 4 and Figure 5), imply that OH° radicals preferentially react with MB molecules rather than HFs organic components (PVDF and PVP).

After 168 h (7 days) of UV irradiation, the MB concentration had decreased by approximately 90%. However, no signs of HF degradation were observed until the end of the experiment (384 h, 16 days) which could at first seem surprising since most of the MB had been degraded.

**Figure 7:**
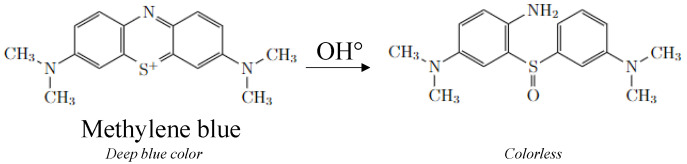
Opening of the central aromatic ring of an MB molecule after electrophilic attack of OH° on the free doublet of heteroatom S.

MB degradation through photocatalysis has been thoroughly described in other works [32,33,34], but those studies fail to mention that the first step of MB oxidation by OH° radicals (cleavage of the central aromatic ring) leads to the discoloration of the molecule (Figure 7) [35]. Therefore, the MB concentration measured with absorbance spectroscopy at 670 nm does not take into account the presence of degradation products in solution after the first oxidation. These biproducts keep on reacting with OH° radicals until total mineralization [32]. The experiment could not be carried out long enough to see HF degradation after all organic molecules had been mineralized. Since the experiment was conducted in static conditions and hydroxyl radicals have an estimated half-life of about 10^–9^ s [36], it is likely that they do not diffuse in the aqueous medium to oxidize MB in the bulk solution. Instead, the reaction must happen near the TiO_2_ NP entrapped in the HFs, where radicals are produced thanks to UV irradiation. MB concentration near the HFs is locally decreased but quickly balanced through the diffusion of MB from the bulk.

These results show that, when irradiating the HFs in a medium that contains an organic pollutant, degradation of HFs’ properties is completely inhibited.

### 3.3. Aging of BSA Pre-Fouled HFs Soaked in UP Water during Irradiation

The previous experiment has shown that the presence of organic matter (MB) near the HFs during UV irradiation would successfully divert OH° radicals from oxidizing the membrane material. We now want to assess if a protein layer on the membrane surface (similar to fouling) would also successfully delay HFs’ degradation by reacting with OH° radicals, while validating the self-cleaning properties of the membrane material. Two methods were used to pre-foul the HFs: with BSA adsorption on one hand and with BSA filtration on the other hand (see Section 2.3). HFs were then exposed to UV irradiation at 1000 µW/cm² in UP water for 384 h. Samples were characterized as described earlier in the study and results are compared with those obtained on non-irradiated HFs (control, black dots) and pristine HFs irradiated in UP water (red dots).

#### 3.3.1. Morphological Degradation

**Figure 8:**
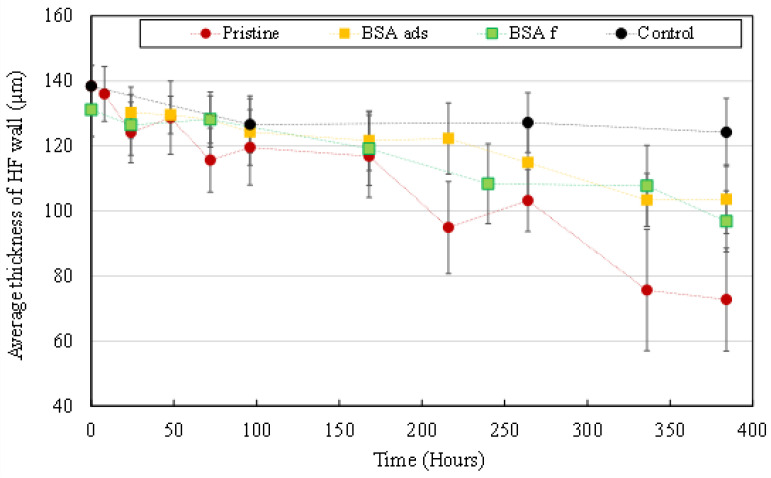
Average thickness of HFs’ wall after UV irradiation at 1000 µW/cm². Pristine HFs (**red dots**), BSA pre-adsorbed HFs (**yellow squares**) and BSA pre-filtrated HFs (**green squares**). Control (**black dots**) = pristine HFs without UV irradiation.

Figure 8 shows the evolution of the average wall thickness of sampled HFs. The presence of a BSA layer on HFs’ surface significantly delays material degradation during UV irradiation. After 384 h of irradiation, the average thickness had decreased by 45% for pristine HFs but only by 29% and 25% for BSA_f_ and BSA_ads_ HFs, respectively. However, there is no significant difference to the average wall thickness between BSA_f_ and BSA_ads_ HFs.

#### 3.3.2. Mechanical Properties’ Evolution

**Figure 9:**
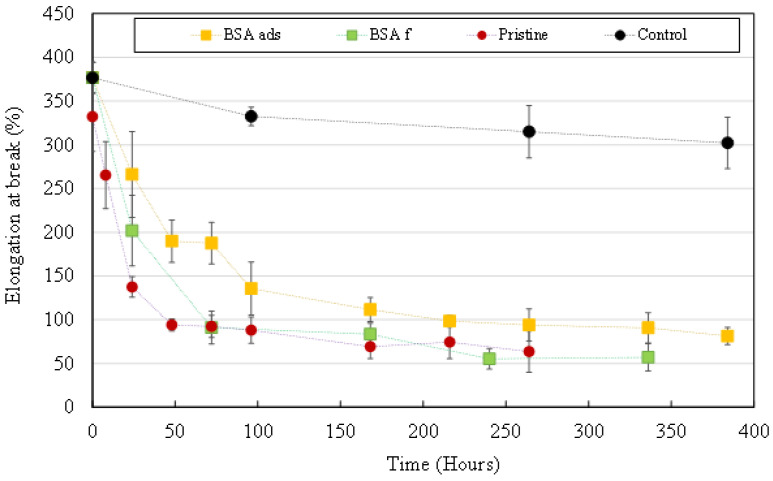
Elongation at break measured on UV irradiated HFs (1000 µW/cm²). Pristine HFs (**red dots**), BSA_ads_ HFs (**yellow squares**) and BSA_f_ HFs (**green squares**). Control (**black dots**) = pristine HFs without UV irradiation.

Figure 9 shows the evolution of the elongation at break values of sampled HFs. During the first hours of UV irradiation, the presence of a BSA layer slightly delays degradation of the mechanical properties of HFs. After 24 h of irradiation, elongation at break had decreased by 64% for pristine HFs but only by 47% and 30% for BSA_f_ and BSA_ads_, respectively. For BSA_f_ HFs, elongation at break results ended up being similar to what was obtained on pristine HFs after 72 h of irradiation. Mechanical properties of BSA_ads_ HFs were slightly more preserved than those of BSA_f_ HFs as the elongation at break values stabilized at around 90% and 55%, respectively during the last 7 days (168 h) of the experiment.

Elongation at break results highlighted the fact that both methods of HFs pre-fouling led to a different extent of protection of the membrane. To further investigate this phenomenon, chemical analysis of HFs’ surface was performed through ATR-FTIR spectroscopy.

#### 3.3.3. Chemical Degradation

**Figure 10:**
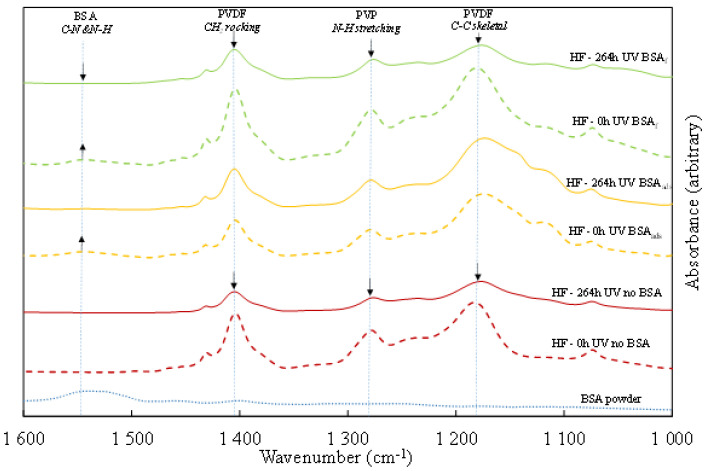
ATR-FTIR spectra for BSA powder (**dotted blue line**), pristine HF (**dashed red line**), 264 h UV irradiated pristine HF (**solid red line**), 0 h UV irradiated BSA_ads_ HF (**yellow dashed line**), 264 h UV irradiated BSA_ads_ HF (**solid yellow line**), 0 h UV irradiated BSA_f_ HF (**green dashed line**), 264 h UV irradiated BSA_f_ HF (**solid green line**).

An ATR-FTIR spectrum was first obtained for BSA powder and it displays a large characteristic peak in the range of 1490–1570 cm^−1^ that corresponds to an amine function of the BSA molecule [37] (Figure 10, dotted blue line). This peak is also visible on the spectra of HFs pre-fouled by BSA before irradiation (Figure 10, dashed yellow and green lines). The spectra obtained on HFs display peaks at 1403 and 1180 cm^−1^ which correspond respectively to the vibration of C–C bonds and CH2 bonds of PVDF. The peak observed at 1291 cm^−1^ corresponds to the stretching of N-H bonds in the PVP molecule [21].

On non-fouled HFs (pristine), peaks associated with PVDF and PVP decrease after 264 h of UV irradiation, which means that polymer bonds are broken by the action of OH° radicals. This is perfectly coherent with observations made in Section 3.3.1 and Section 3.3.2. where significant morphological and chemical degradation was noticed, which led to the embrittlement of the HFs and significant losses to their mechanical properties.

Spectra obtained on BSA_ads_ HFs before and after UV irradiation display similar PVDF and PVP peak height, meaning that the chemical structure of HFs was preserved from OH° attacks. As the peak associated with BSA disappeared from the BSA_ads_ spectra after UV irradiation, we conclude that HFs self-cleaning was successful.

Comparison of spectra obtained on BSA_f_ HFs before and after irradiation also show the disappearance of the peak associated with BSA. However, peaks associated with PVDF and PVP show a significant decrease after irradiation. This means that, even if the degradation of the BSA layer could have slightly delayed the action of OH° radicals on polymer bonds, the protective effect of the BSA layer was not enough to preserve the -membrane material.

Discussing and comparing the results obtained with BSA_ads_ and BSA_f_ HFs could not be performed on the basis of the amount of BSA present on HFs, as this value could not be calculated with an acceptable accuracy for BSA_ads_ HFs (neither by mass balance in the soaking protein solution, nor by FTIR analysis). However, we can extrapolate the phenomena that occurred during HF pre-fouling with both methods. When pre-fouling HFs by filtration of a 2 g/L BSA solution in a dead-end system, BSA molecules (molecular weight ≈ 66 kDa, mean radius: 3.5 nm) are pushed inside membrane pores (average pore diameter 49 ± 0.3 nm). Fouling happened inside HFs’ wall and water permeability was decreased by 33% (from 283 L/h/m²/bar for pristine membrane to 189 L/h/m²/bar for BSA_f_ HFs). When pre-fouling HFs by adsorption through soaking the membrane in a 10 g/L BSA solution, BSA molecules were exclusively adsorbed on HFs’ external surface and did not penetrate inside the pores. Consequently, water permeability was not affected.

When irradiating HFs with UV light, only the external TiO_2_ NP are activated and OH° radicals are produced only near HFs’ external surface. We can therefore hypothesize that BSA molecules which are adsorbed on HFs’ surface are easily degraded by locally produced OH°, but that these radicals do not reach BSA molecules inside the pores. If we assume that a similar amount of BSA got deposited on BSA_ads_ and BSA_f_ HFs, these results seem to show that external BSA layer on BSA_f_ HFs was not enough to protect the membrane from OH° attacks as most BSA molecules got pushed inside the pores during dead-end filtration.

In real-life working conditions, with a complex effluent composed of organic matter and various organic pollutants, the membrane should be preserved. At this step of the study, it is still difficult to extrapolate without further pilot-scale experiments that optimize the irradiation conditions that may be continuous or discontinuous.

## 4. Conclusions

In this study, the effect of UV irradiation on aging of PVDF-PVP-TiO_2_ HF membranes was thoroughly investigated. Pristine HFs irradiated between 250 and 1000 µW/cm² in UP water showed signs of morphological degradation from the seventh day of irradiation (168 h). HFs irradiated at 1000 µW/cm² had a wall thickness reduced by 50% after 16 days of irradiation (384 h), with emergence of macrovoids and formation of cracks on membrane surface. Mechanical properties of HFs were affected from the first hours of irradiation for all tested power levels. In the case of samples irradiated at 500 and 1000 µW/cm² for more than 264 h, we observed a strong embrittlement phenomenon that made it impossible to correctly perform tensile strength measurements.

Experiments performed in a soaking medium containing MB as a model pollutant showed that OH° radicals responsible for membrane degradation would preferentially react with MB instead of the membrane material, thus preserving HFs morphological and mechanical properties. Experiments performed with BSA pre-fouled HFs showed that the presence of fouling would also delay aging since OH° radicals would react with the BSA layer as well. However, when BSA molecules were mostly inside the porosity of the membrane and not on its external surface, OH° could not react with them as they have a half-life of about 10–9 s and are produced on the external side of HFs, where UVA light reaches TiO_2_ NP.

These results, obtained in a lab-scale context of accelerated aging, highlight the need to finely monitor the amount of UV irradiation received by the membranes according to the cleaning needs and organic matter concentration in the effluent. Ultimately, the aim for industrial use of these membranes is to maintain 70% to 80% of their mechanical properties during their lifespan. Continuous filtration tests with pulsating UV irradiation and protein solutions (BSA and humic acid) are now in progress to assess the membrane low fouling and self-cleaning performance in closer-to-industrial-scale conditions.

## Figures and Tables

**Figure A1:**
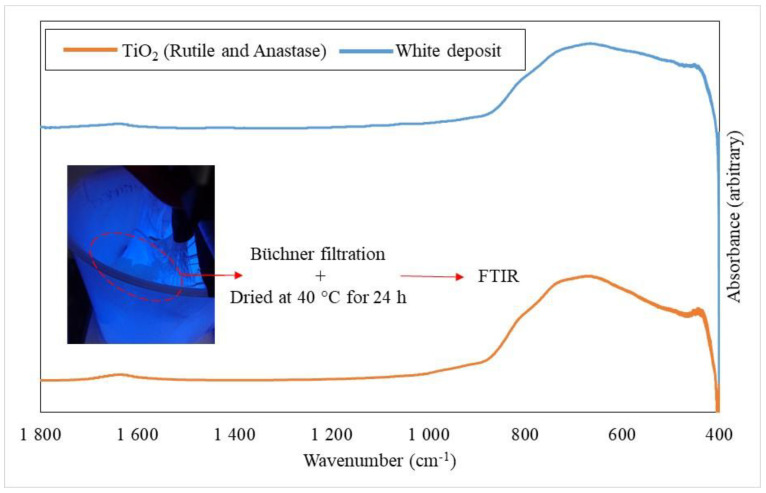
FTIR spectra of commercial TiO_2_ used in the production of the membrane (**orange**) and of the dried white deposit observed in the soaking medium (**blue**).

## Data Availability

The data presented in this study are available in the article.
